# Computed Tomography Imaging of Primary Lung Cancer in Mice Using a Liposomal-Iodinated Contrast Agent

**DOI:** 10.1371/journal.pone.0034496

**Published:** 2012-04-02

**Authors:** Cristian T. Badea, Khannan K. Athreya, Gabriela Espinosa, Darin Clark, A. Paiman Ghafoori, Yifan Li, David G. Kirsch, G. Allan Johnson, Ananth Annapragada, Ketan B. Ghaghada

**Affiliations:** 1 Center for In Vivo Microscopy, Duke University Medical Center, Durham, North Carolina, United States of America; 2 University of Texas Medical School at Houston, The University of Texas Health Sciences Center at Houston, Houston, Texas, United States of America; 3 The Edward B. Singleton Department of Pediatric Radiology, Texas Children's Hospital, Houston, Texas, United States of America; 4 School of Biomedical Informatics, The University of Texas Health Sciences Center at Houston, Houston, Texas, United States of America; 5 Department of Radiation Oncology, Duke University Medical Center, Durham, North Carolina, United States of America; 6 Department of Pharmacology and Cancer Biology, Duke University Medical Center, Durham, North Carolina, United States of America; University of Texas, M.D. Anderson Cancer Center, United States of America

## Abstract

**Purpose:**

To investigate the utility of a liposomal-iodinated nanoparticle contrast agent and computed tomography (CT) imaging for characterization of primary nodules in genetically engineered mouse models of non-small cell lung cancer.

**Methods:**

Primary lung cancers with mutations in K-ras alone (Kras^LA1^) or in combination with p53 (LSL-Kras^G12D^;p53^FL/FL^) were generated. A liposomal-iodine contrast agent containing 120 mg Iodine/mL was administered systemically at a dose of 16 µl/gm body weight. Longitudinal micro-CT imaging with cardio-respiratory gating was performed pre-contrast and at 0 hr, day 3, and day 7 post-contrast administration. CT-derived nodule sizes were used to assess tumor growth. Signal attenuation was measured in individual nodules to study dynamic enhancement of lung nodules.

**Results:**

A good correlation was seen between volume and diameter-based assessment of nodules (R^2^>0.8) for both lung cancer models. The LSL-Kras^G12D^;p53^FL/FL^ model showed rapid growth as demonstrated by systemically higher volume changes compared to the lung nodules in Kras^LA1^ mice (p<0.05). Early phase imaging using the nanoparticle contrast agent enabled visualization of nodule blood supply. Delayed-phase imaging demonstrated significant differential signal enhancement in the lung nodules of LSL-Kras^G12D^;p53^FL/FL^ mice compared to nodules in Kras^LA1^ mice (p<0.05) indicating higher uptake and accumulation of the nanoparticle contrast agent in rapidly growing nodules.

**Conclusions:**

The nanoparticle iodinated contrast agent enabled visualization of blood supply to the nodules during the early-phase imaging. Delayed-phase imaging enabled characterization of slow growing and rapidly growing nodules based on signal enhancement. The use of this agent could facilitate early detection and diagnosis of pulmonary lesions as well as have implications on treatment response and monitoring.

## Introduction

Lung cancer is the leading cause of cancer death (∼28%) in both men and women and the number of deaths are expected to increase 50% by 2020 worldwide [1]. In order to reduce mortality rates, the focus on lung cancer management has shifted to early detection and personalized cancer therapy [2]. In a recent study, computed tomography (CT) screening of patients at high risk reduced lung cancer deaths by 20% [3] demonstrating the potential benefit of screening for early stage lung cancer. However, surveillance of suspected lung nodules frequently requires longitudinal follow-up to evaluate changes in nodule size and growth rate. A majority of these cases require multiple follow-up CT scans, ranging up to two years, before a diagnosis of malignancy is made [4]. Advances in imaging techniques that improve characterization of pulmonary nodules could have a substantial impact on patient management and economic burden of lung cancer.

Novel imaging techniques that can take advantage of differences in tumor morphology between malignant and benign nodules are being evaluated. Dynamic contrast-enhanced (DCE)-CT imaging has been evaluated for differentiation of benign and malignant tumors based on nodule perfusion and tumor vessel permeability [5], [6]. While promising, the use of conventional contrast agents present challenges in quantitative perfusion analysis due to rapid leakage into the extravascular space, even during first pass imaging. Furthermore, the molecular nature of iodinated contrast agents, similar in size to Magnetic Resonance (MR) contrast agents, makes them less sensitive to changes in vascular morphology that occur at the nano and micro scales [7], [8], [9]. Macromolecular and nanoparticle-based imaging agents could potentially provide a more accurate measurement of nodule perfusion and vessel permeability. In a recent pre-clinical study, the use of a nanoparticle-based, liposomal-iodinated CT contrast agent for differentiation of tumors based on their growth-rate was demonstrated using 2D clinical mammography in a rat model of mammary adenocarcinoma [10]. The study demonstrated that rapidly growing tumors showed increased vascular permeability to nanoparticle contrast agents when compared to slow growing tumors. In this study, we therefore sought to evaluate the utility of the liposomal-iodinated CT contrast agent for characterization of slow growing and rapidly growing pulmonary nodules in genetically engineered mouse models of primary non-small cell lung cancer.

## Materials and Methods

### 1. Ethics statement

All animals were handled in accordance with good animal practice as defined by the relevant national and/or local animal welfare bodies, and all animal work was approved by the Institutional Animal Care and Use Committee (IACUC) of Duke University Medical Center. The Duke University Medical Center animal management program is accredited by the American Association for the Accreditation of Laboratory Animal Care and meets National Institute of Health standards as set forth in the “Guide for the Care and Use of Laboratory Animals” (DHHS Publication No. (NIH) 85–23, Revised 1985). The institution also accepts as mandatory the PHS “Policy on Humane Care and Use of Laboratory Animals by Awardee Institutions” and “NIH Principles for the Utilization and Care of Vertebrate Animals Used in Testing, Research and Training”.

### 2. Fabrication of liposomal CT contrast agent

Liposomal-iodinated CT contrast agent was prepared using methods described previously [7]. Briefly, a lipid mixture (150 mmol/L) consisting of 1,2-dipalmitoyl-sn-glycero-3-phosphocholine (DPPC), cholesterol, and 1,2-distearoyl-sn-glycero-3-phosphoethanolamine-N-[methoxy (polyethylene glycol)-2000] (DSPE-MPEG 2000) in a 55∶40∶5 molar ratio was dissolved in ethanol. The ethanol solution was hydrated with iodixanol solution (550 mg I/mL) and then sequentially extruded on a Lipex Thermoline extruder (Northern Lipids, Vancouver, British Columbia, Canada) to size the liposomes to ∼100 nm. The resulting solution was diafiltered using a MicroKros® module (Spectrum Laboratories, CA) to remove un-encapsulated iodixanol. The size distribution of liposomes in the final formulation was determined by dynamic light scattering (DLS) using a Malvern Zetasizer Nanoseries (Malvern Instruments, Worcestershire, UK) at 25°C. The iodine concentration in the final liposomal solution was quantified by spectrophotometry (Abs at 245 nm). The final iodine concentration in the PEGylated liposomal-iodine formulation was 120 mg/mL. The average liposome size was 118±20 nm and the poly-dispersity index was less than 0.15.

### 3. In vivo studies

#### i. Primary lung cancer models and tissue processing

Primary lung tumors were developed as described previously [11], [12], [13]. Two primary lung cancer models were developed in this study: LSL-Kras^G12D^;p53^FL/FL^ mice with expression of oncogenic Kras^G12D^ and deletion of p53 following intranasal infection with Adeno-Cre; and Kras^LA1^ mice with expression of only oncogenic Kras^G12D^ following spontaneous intra-chromosomal recombination of the latent Kras allele. The LSL-Kras^G12D^;p53^FL/FL^ animals were used for the imaging study at 12 weeks post Adeno-Cre infection. All animals were imaged at 24–30 weeks of age. A total of ten animals (five per group) were used for the imaging study.

After the final imaging session, the animals were sacrificed and perfused with phosphate buffered saline. The lungs were extracted, snap frozen in liquid nitrogen and then stored at −80°C. Hematoxylin and Eosin (H&E) staining was performed on the tissue sections to survey tumor morphology.

#### ii. Micro-CT setup and imaging study

A custom-built dual-source-detector micro-CT imaging system was used for the study [14]. Only one X-ray source and detector, were used for imaging and a scan took about 7 minutes to complete. The animals were scanned while free breathing under anesthesia using 2–3% isoflurane delivered by nose-cone setup. Prospective cardio-respiratory gating was used to minimize effects of animal respiratory and cardiac motion during scans [15]. A pneumatic pillow positioned on the animals' thorax connected to a pressure transducer was used to provide an electrical signal that correlated with the breathing motion. ECG pads were used to acquire the ECG signal. A LabVIEW application read both the respiratory and ECG signals and provided a Transistor–Transistor Logic pulse required for triggering the X-ray tube and detector in end-expiration and on the R peak of the ECG cycle. The scanning parameters were: 80 kvp, 160 mA, 10 ms/exposure. A total of 300 views were acquired over 360° rotation. The dose associated with a single scan was 8 cGy. Volumes were reconstructed using the Feldkamp algorithm [16] in a matrix of 512×512×512 at 88 µm isotropic voxel size.

Longitudinal micro-CT imaging was done in all animals. A pre-contrast, baseline scan was acquired. Subsequently, the liposomal contrast agent was intravenously injected over 2 minutes via the tail vein at a volume dose of 16 µL/gm (∼1920 mg Iodine/kg dose) of body weight. Post-contrast scans were then acquired immediately (0 hr time-point) and at day 3 (72 hr) and day 7 (168 hr) after administration of the liposomal contrast agent.

### 4. Image data analysis

Data analysis of all image sets was performed in Osirix (v.3.6 64-bit). Volume and maximum diameter measurements were performed on individual lung nodules. A minimum of four nodules were analyzed in each animal except for one animal in the benign group that only showed one detectable nodule. Lung nodules were manually segmented by drawing regions of interest in the axial plane. The largest nodule cross-section was manually identified in the axial plan and the nodule diameter was determined using the line tool in Osirix. The lung nodules were then assigned into one of the following three groups based on the nodule diameter (x in mm) measured in the axial plane: Group 1 (1.0<x≤1.5), Group 2 (1.5<x≤2.5) and Group 3 (2.5<x≤3.5). Nodules smaller than 1 mm were not analyzed because they represented a very large number of nodules with extremely rapid volume changes that were convoluted by animal positioning and which hindered tumor matching in the imaging sets at different time points.

Relative changes in nodule size were computed as:
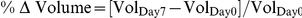
Vol_Day0_ is the average of pre-contrast and 0 hr post-contrast nodule volume; Vol_Day7_ is the nodule volume on day 7 following contrast injection.

The blood clearance of contrast agent was determined by measuring signal attenuation in a major vessel, the descending aorta. For the nodules, average signal attenuation was computed over the entire volume. ROIs were drawn at three different locations for descending aorta. Signal attenuation was presented as average values and standard deviations reported in Hounsfield units (HU).

The differential signal enhancement in each nodule was computed as:
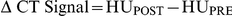
where, HU_POST_ is the average nodule signal, in Hounsfield units (HU), immediately post-contrast (0 hr) or day 3 or day 7 and HU_PRE_ is the average nodule signal, in Hounsfield units (HU), in pre-contrast scan.

The fractional blood volume (FBV), expressed as percentage, in each nodule was determined using the pre-contrast and the post-contrast 0-hr datasets according to the equation [17]:
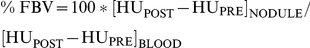
Since residual blood-pool signal was detected on day 7, the differential signal enhancement for the nodule was corrected to eliminate the blood volume component of the overall nodule signal using the following equation:
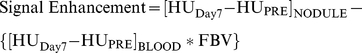
Osirix (v-3.6, 64-bit) and ImageJ (v-1.41o) were used for visual representation.

## Results

**Figure 1:**
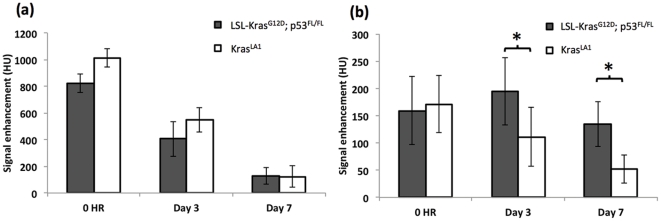
Dynamic signal enhancement in (a) blood and (b) nodules, in the two primary lung cancer models. Errors bars represent standard deviations (* indicates p<0.05).

**Figure 2:**
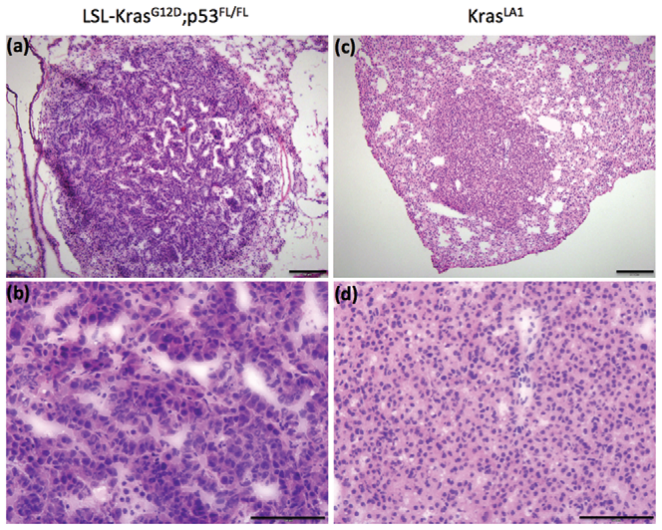
Hematoxylin and eosin staining showing characteristics of high-grade lung cancer in LSL-Kras^G12D^;^p53FL/FL^ mice with pleomorphic nuclei (a,b) and low-grade Kras^LA1^ lung tumors in Kras^LA1^ mice with regular nuclei and minimal cytologic atypia (c,d). Images were acquired at 10× (a,c) and 40× magnification (c,d). Scale bars: 200 um in a and c; 100 um in b and d.

A high blood signal enhancement was obtained immediately post-administration of liposomal contrast agent (Figure 1a). The signal decayed gradually over time and by day 7, the majority of liposomal contrast agent had cleared from systemic circulation as evident by a reduction in blood attenuation signal. Previous imaging studies using the liposomal contrast agent in mice have reported a blood half-life of approximately 41 hours [7]. Dynamic analysis of lung nodules demonstrated signal enhancement immediately post-contrast (0 hr) indicating high blood volume in the nodules (Figure 1b). The Kras^LA1^ model showed gradual decrease in signal enhancement over time, similar to observed trend for clearance of the contrast agent from systemic circulation. On day 3 and day 7, the nodules in LSL-Kras^G12D^;p53^FL/FL^ model showed significantly higher signal enhancement (p<0.05) compared to nodules in the Kras^LA1^ model. Histological analysis of both the primary lung cancer models demonstrated characteristics similar to those described previously (Figure 2) [11], [12], [13].

**Figure 3:**
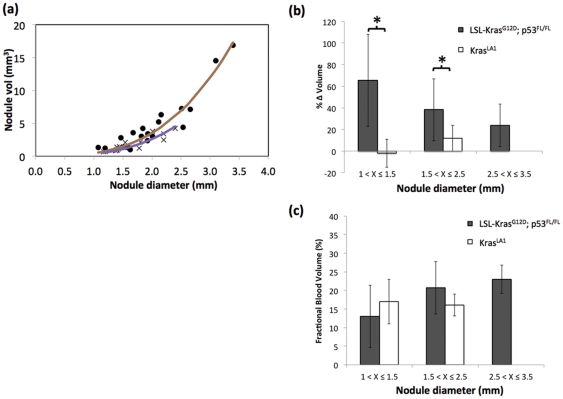
Correlation between nodule volume and diameter in LSL-Kras^G12D^;p53^FL/FL^ (•) and Kras^LA1^ (×) models. (**a**)**.** Solid lines indicate cubic fit to the data points. An R^2^ value of 0.93 and 0.81 was obtained for LSL-Kras^G12D^;p53^FL/FL^ and Kras^LA1^ models, respectively. Percentage change in nodule volume in the two lung cancer models as a function of nodule diameter (* indicates p<0.05) (b). CT-derived fractional blood volume as a function of nodule diameter (c).

Analysis of pulmonary nodules demonstrated good correlation between diameter-based and volume-based measurements for both lung cancer models (r^2^>0.8) (Figure 3a). The nodule volumes were measured using micro-CT on day 0 and day 7 post-administration of the contrast agent. During the imaging period, the nodules in LSL-Kras^G12D^;p53^FL/FL^ mice demonstrated significant increase (p<0.05) in volume compared to nodules in Kras^LA1^ mice, indicating higher growth rate (Figure 3b). The Kras^LA1^ mice did not show nodules larger than 2.5 mm, most likely due to the slow-growing nature of this model. The blood-pool property of the liposomal contrast agent also enabled determination of fractional blood volume. No significance differences in fractional blood volume were seen between the two lung cancer models (p<0.05) (Figure 3c).

**Figure 4:**
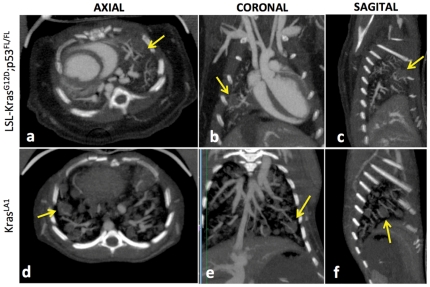
Orthgonal thick slab maximum intensity projection (MIP) images demonstrating visualization of nodule blood supply in the lung cancer models. The images were acquired immediately after administration of liposomal contrast agent.

Both type of lung nodules showed higher attenuation immediately after administration of liposomal contrast agent, indicating high tissue perfusion. The high blood-pool attenuation enabled visualization of vascular network associated with the lung nodules (Figure 4). Large blood vessels at the surface of the nodules were observed in both models. The small feature size made it difficult to probe the vascular structures within the nodule.

**Figure 5:**
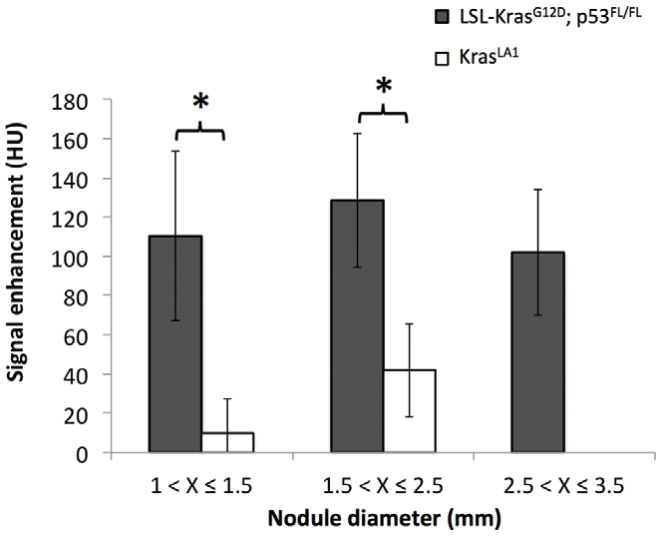
Differential signal enhancement on day 7 in the two primary lung cancer nodules. (* indicates p<0.05).

**Figure 6:**
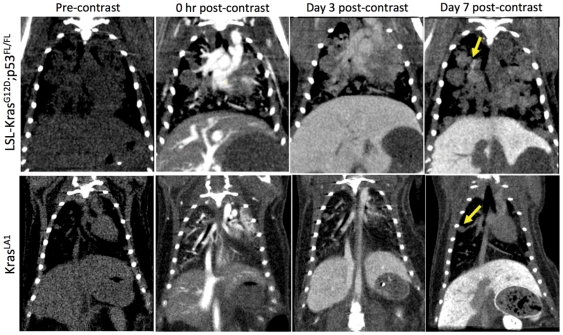
Representative coronal micro-CT images of LSL-Kras^G12D^;p53^FL/FL^ and Kras^LA1^ mice before liposomal contrast-agent injection and at 0 hr, Day 3 and Day 7 post-contrast injection. Note the differential enhancement of tumors at Day 7 post-contrast time point in the LSL-Kras^G12D^;p53^FL/FL^ lesions only.

Average nodule signal attenuation was measured at baseline and day 7 post-administration of the liposomal contrast agent. Signal enhancement in the nodules was determined relative to baseline (Figure 5). The lung nodules in LSL-Kras^G12D^;p53^FL/FL^ mice showed significantly higher signal enhancement compared to the nodules in Kras^LA1^ mice (p<0.05). The longitudinal aspect of this study also enabled facile imaging of the delayed tumor enhancement in the LSL-Kras^G12D^;p53^FL/FL^ mice (Figure 6).

## Discussion

Early detection of primary lung cancer can lead to improved patient survival. Angiogenesis, one of the hallmarks of solid tumors, involves the development of new blood vessels. Unlike normal vessels, tumor-associated vessels have abnormal and ‘leaky’ architecture, exemplified by the presence of large fenestrations within the endothelial lining that enable not only small molecules and particulate matter to extravasate into the interstitial region but also facilitate tumor cells to escape into the systemic circulation [18]. Non-invasive advanced imaging techniques that can exploit differences in tumor micro-environment can play an important role in early detection of cancer.

Several modalities have been used for pre-clinical imaging in mouse models of lung cancer [11], [19]. Among these, CT and MRI, which are routinely used in the clinic, provide high-spatial resolution for assessing vascular and morphological changes. Nuclear imaging techniques such as Positron Emission Tomography (PET) and Single Positron Emission Computed Tomography (SPECT), also used clinically for diagnosis and therapeutic monitoring of lung cancer, provide high-contrast sensitivity but with relatively low-spatial resolution. Optical imaging techniques, such as fluorescence tomography and bioluminescence, which also provide high sensitivity, have primarily been used pre-clinically to study lung cancer growth as well as to monitor treatment response [20], [21]. However, the light-based modalities are plagued by low spatial resolution and limited tissue penetration. They are performed in combination with micro-CT to provide not only anatomical reference but also improve image reconstruction. Primary lung cancers in mice, unlike many other tumor types, are challenging to image with high-resolution due to cardiac and respiratory motion artifacts and small tumor sizes. In this work, we investigated the use of a liposomal-iodinated contrast agent and micro-CT for characterization of primary lung cancers in mice. The use of prospective cardio-respiratory gating enabled acquisition of high quality tomographic and isotropic images at voxel dimension of 88 µm. Motion challenges imposed by lung imaging in rodents were overcome with minimally-invasive procedures i.e., they required no intubation and mechanical ventilation. The associated radiation dose of 0.24 Gy accumulated over three imaging time points was in the typical range reported in the literature [22] and therefore is not expected to play a role in the outcome of results of our imaging study. We note however that the radiation dose in preclinical studies with micro-CT is much higher than in clinical studies. This is because a higher resolution is required in micro-CT and this could be only achieved using more radiation.

The genetically engineered mouse models of primary lung cancer used in this study, have previously been characterized and tested for evaluating chemotherapies and radiation treatment [11], [23]. The LSL-Kras^G12D^;p53^FL/FL^ model, which has mutations in Kras and p53, results in the generation of aggressive primary adenocarcinomas. The Kras^LA1^ model, which has mutations in Kras only, results in the development of primary adenomas which can progress to low grade adenocarcinomas. The LSL-Kras^G12D^;p53^FL/FL^ model showed characteristics of rapid growth consistent with high grade adenocarcinomas that were consistent with previous studies [11], [23]. A strong correlation (R^2^>0.8) was observed between nodule diameter and volume for both cancer models. Concerns have been raised about Response Evaluation Criteria in Solid Tumors (i.e. RECIST)-based response assessments, in part because tumors do not always expand or contract uniformly; changes in line lengths represent only a small fraction of the available information in the images [24]. However, because the two measures correlated well in this study and the size-based analysis is a commonly used technique in the clinic, subsequent analysis of nodule growth and signal enhancement was studied by classifying nodules based on their size.

As demonstrated in this study, the liposomal-iodinated contrast agent provides two methods for characterizing solid tumors. During early-phase imaging, which occurs within a few hours post-administration of the contrast agent, the agent is primarily distributed in the vascular compartment with negligible extravasation into the tumor tissue. As a result, visualization of tumor vasculature and blood supply is achieved, thus enabling assessment of relative blood volume in tumors. As time progresses, the liposomal-iodinated nanoparticles extravasate into the tumor tissue via the enhanced permeation and retention effect resulting in tumor signal enhancement. Consequently, during delayed-phase imaging, which occurs over several days, the liposomal contrast agent enables imaging and differentiation of tumor tissue.

The higher signal enhancement observed in the LSL-Kras^G12D^;p53^FL/FL^ model suggest increased accumulation and therefore enhanced vascular permeability to the nanoparticle contrast agent. Similar phenomenon of increased vascular permeability to nanoparticles has also been observed in other highly aggressive tumor models [10]. Furthermore, a recent study also showed, very elegantly, the changes in tumor vascular permeability to nanoparticles as tumor transition from pre-malignant status to a malignant status [25]. Taken together, these findings suggest that rapidly growing tumors take up more liposomal contrast agent than slow growing tumors. These could have important clinical implications as it may enable differentiation and classification of tumors based on their malignancy potential. However, it is important to note that nodules, even malignant ones, seen in the clinical setting have relatively slower growth rates compared to those seen in this study. Thus, such evaluation will ultimately have to be made in the clinic to determine potential of this method for effective characterization and staging of tumors. The increased uptake of liposomal contrast agent in rapidly growing tumors suggest that one could use nano-carriers to deliver high payloads of chemotherapeutics or genetic materials within these nodules, simply via passive extravasation. Furthermore, the ability to highlight potential malignant nodules could also facilitate biopsy as well as accurately delineating tumors margins using CT imaging for radiotherapy or surgical removal.

As recently shown, targeted delivery of imaging agents and therapeutics to lung tumors is possible and could provide early detection and increased therapeutic efficacy against cancer [26]. Freedman et al [27] have recently used a targeted immunoliposome complex for MRI imaging of lung tumors. The versatile nature of liposomal platform and its precedent for use in the clinic has seen a continued interest in this area resulting in the development of non-targeted and targeted imaging agents for use in a variety of imaging modalities [28], [29], [30].

Although possible, we have not used targeted liposomes in this study. Instead we have shown that sufficient differential enhancement of lung tumors can be obtained based on passive accumulation of liposomes. The current study also has some limitations. The iodine dose used in this study was 5–10 fold higher than the iodine dose used routinely in the clinic for dynamic CT imaging of lung tumors. The high dose was necessary to overcome relatively higher noise levels (6–10 fold) in micro-CT compared to clinical CT and to visualize microvascular structures. Consequently, a longer waiting period was required to allow blood clearance of a majority of the contrast agent. While the dose of contrast agent can be reduced, the high noise levels associated with micro-CT systems (>65 HU) that arise from high spatial resolution present challenges in quantitative evaluation of small features. A high lipid dose, in order to deliver high iodine dose, was also used in this study. While such doses cannot be used in the clinic, we believe that the low noise levels in clinical CT scanners and the large feature size would enable reduction in contrast agent, and a corresponding lipid, dose. Previous studies have demonstrated imaging of clinical-size lesions in rabbit model using liposomal contrast agent administered at an iodine dose comparable to those used in the clinic [31].

The study also provided insights into quantitative perfusion imaging using a blood pool contrast agent and CT imaging. However, routine analysis is challenging in rodent studies due to small feature size and high noise levels on micro-CT scanners. Advanced methods that can reduce noise levels, such as iterative reconstruction techniques [32], [33], coupled with increased contrast-sensitive dual energy imaging techniques [34] may help in achieving these goals. The success of such imaging procedures would provide new opportunities to assess the efficacy of anti-angiogenic therapies in pre-clinical cancer models.
